# Bone wax reduces blood loss after total hip arthroplasty: a prospective, randomized controlled study

**DOI:** 10.3389/fmed.2023.1246733

**Published:** 2023-09-01

**Authors:** Hao Li, Chao Huang, Zi-Chuan Ding, Zun-Han Liu, En-Ze Zhao, Zong-Ke Zhou

**Affiliations:** ^1^Department of Orthopaedics, West China Hospital, Sichuan University, Chengdu, China; ^2^Department of Joint Surgery, First Affiliated Hospital of Sun Yat-sen University, Guangzhou, China; ^3^Department of Sports Medicine Center, the First Affiliated Hospital of the Army Military Medical University, Chongqing, China

**Keywords:** bone wax, total hip arthroplasty, postoperative blood loss, blood-conserving management, transfusion

## Abstract

**Background:**

Previous studies have demonstrated the efficacy of bone wax in reducing blood loss in various orthopedic surgeries. However, the effect of bone wax on total hip arthroplasty (THA) remains unclear. The objective of this study was to assess the efficacy of bone wax in THA.

**Methods:**

We enrolled 104 patients in this randomized controlled trial. These patients were randomized (1:1) to either the bone wax or control group. The primary outcome was total blood loss after THA. The secondary outcomes included serum hemoglobin (Hb) level, change in Hb level, lower limb diameters on the first and third postoperative day (POD), range of motion at discharge, length of postoperative hospital stay, and adverse events.

**Results:**

Patients in the bone wax group had significantly lower total blood loss on PODs 1 and 3 (*p* < 0.05). Moreover, patients in the bone wax group performed better in terms of postoperative serum Hb level, change in Hb level on PODs 1 and 3, and length of postoperative hospital stay (all *p* < 0.05). Patients in the bone wax group did not experience any bone wax-related adverse events.

**Conclusion:**

Bone wax administration in THA significantly reduced perioperative blood loss. Therefore, bone wax is promising for optimizing blood-conserving management protocols in THA.

**Clinical trial registration:**

[https://clinicaltrials.gov/], identifier [ChiCTR2100043868].

## Introduction

Total hip arthroplasty (THA) is a common orthopedic surgery used to treat end-stage hip joint diseases (1, 2). However, THA may result in substantial perioperative blood loss and necessitate transfusion, which could further lead to higher morbidity and mortality, increased length of hospital stay, and delayed functional recovery (3–5). Therefore, perioperative blood-conserving management in THA has always been a major topic in related research efforts (6).

Substantial bleeding in THA is mainly caused by procedures for the dissection of soft tissue, bone resection, and opening of the femoral medullary canal (6). Hemostasis of soft tissue can be controlled using a bipolar coagulator and maintained by mechanical compression (7). However, continuous bleeding from the bone is conspicuous and difficult to manage because the bleeding sites are always deeply seated and difficult to find (7). During surgery, waiting for natural hemostasis to occur is not an option; therefore, additional hemostatic methods are required to effectively and immediately stop bone bleeding (7–9).

Bone wax is a well-known topical hemostatic agent primarily composed of beeswax and softening agents such as petroleum jelly or a mixture of paraffin and isopropyl palmitate (10, 11). Its hemostatic action relies on the mechanical properties of trabecular vascularization of bone to seal the bleeding site and prevent blood flow from broken blood vessels into the bone to promote clot formation (6, 7). Because blood vessels are commonly distributed in the cortical and cancellous bones and osseous hemorrhage from the naked bone section and the femoral canal uncovered by the prostheses is a major source of blood loss in THA, the clinical application of bone wax in THA should be beneficial (11–14).

However, to the best of our knowledge, studies on the effect of bone wax for reducing blood loss in the arthroplasty are very rare (6, 11). Based on the hypothesis that bone wax significantly reduces blood loss in THA and leads to further positive postoperative outcomes, we conducted a prospective, randomized controlled study to determine whether bone wax optimizes perioperative hemostasis protocols in THA.

## Materials and methods

### Study design

A prospective single-center randomized controlled clinical trial was performed. This study was approved by the institutional review board of our institution and registered in the Chinese Clinical Trial Registry (ChiCTR2100043868). All recruited patients voluntarily participated in this study, and signed the informed consent and research authorizations. This study conformed to the unified randomized clinical trial guidelines, and complied with the Declaration of Helsinki (15).

### Patient recruitment

The inclusion criteria included age over 18 years old, an American Society of Anesthesiologists (ASA) class of I, II, or III, and end-stage hip joint diseases reaching surgical indications for primary THA. The exclusion criteria included a history of bone wax allergy, acute infection of the hip joint, inflammatory arthritis, recent treatment of malignant disease, hemoglobin (Hb) of less than 11 g/L, major previous ipsilateral hip arthroplasty or open surgery, bleeding disorders, impaired coagulation function, and high-risk medical comorbidities.

The necessary sample size calculation was based on a previous research (6). Fifty-two patients in each group were required for a two-sided hypothesis study at a power of 0.90, an alpha level of 0.05, and a dropout rate of 10% for detecting a 100-mL difference in blood loss between groups.

Participants were randomly allocated (1:1) to either the bone wax group or the control group. Randomization was concealed from the researchers and patients and assigned using sealed envelopes after hospitalization. A sealed and opaque envelope was prepared in advance to store a randomized grouping plan for this study. After arranging a sickbed for an eligible patient and excluding surgical contraindications, an independent researcher opened the randomized envelope according to the order in which the patients were enrolled to determine the grouping. Participants and the main investigator did not know the grouping situation until the end of the data analyses.

### Surgical procedure and blood-conserving managements

**Figure 1:**
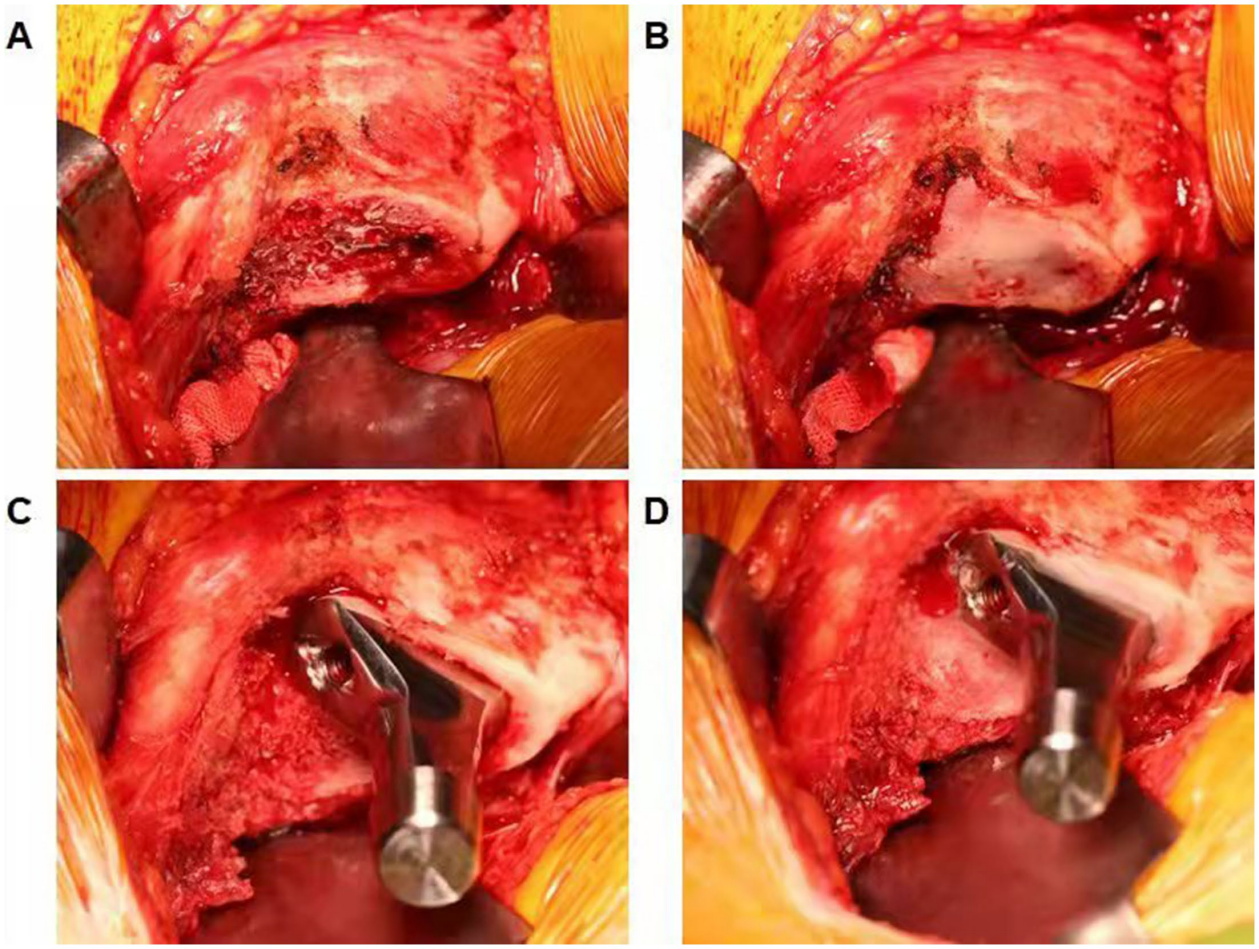
**(A)** Demonstrating the femoral canal after osteotomy of the femoral neck. **(B)** Bone wax was used to seal the femoral canal and removed before opening the medullary canal. **(C)** Demonstrating exposed cancellous bone surface around the femoral prostheses. **(D)** Bone wax was applied on the exposed cancellous bone surface around the femoral prostheses.

Surgery was performed under general anesthesia by the same senior doctor who has performed over 150 THAs per year. The patients were placed in the lateral decubitus position. The hip skin incision was made through the fascia over the greater trochanter. Then, the gluteus maximus was split, and the external rotators were detached. The posterolateral approach was performed as described previously (16). The surgeon was informed of the randomization after the incision in the hip joint capsule. The Corail®cementless stem (DePuy Synthes) was used in all participants. In the bone wax group, bone wax was applied to seal the femoral canal after osteotomy of the femoral neck and removed before opening the medullary canal. After the implant of the femoral stem was held in place, bone wax was used on the exposed cancellous bone surface around the femoral prostheses (Figure 1). Bone wax was not used in the control group. The surgeon utilized electrocautery to achieve hemostasis throughout the procedure, and all patients underwent an additional standardized hemostasis strategy, that is, 20-mg/kg intravenous tranexamic acid bolus 5 min before the incision (17). No drain was used during surgery, and a unified thromboembolic prophylaxis protocol was applied for all participants. Enoxaparin 20 mg was administered subcutaneously 8 h after surgery and then enoxaparin 40 mg once daily from the first day after surgery to hospital discharge, when oral apixaban 5 mg daily was prescribed until 2 weeks after surgery (18). Lower-extremity strength training was performed before surgery, and early mobilization was performed after anesthesia resolution. All patients were evaluated daily for symptomatic deep vein thrombosis (DVT) and pulmonary embolism (PE). If a patient had any venous thromboembolism symptoms during the 3-month follow-up period, a diagnostic Doppler ultrasound of both lower limbs and computed tomography (CT) pulmonary arteriography were performed immediately.

### Outcome measurements

The following preoperative demographics and characteristics were recorded: age, sex, height, weight, body mass index, ASA status, affected side, diagnosis, serum Hb level, blood volume, lower limb diameters, range of motion (ROM), and Harris hip score. The primary outcome was total blood loss after THA, which was calculated using the Gross equation: total blood loss (mL) = BV × (Hct_pre_ − Hct_post_), where BV (mL) represents the patient’s blood volume before surgery, Hct_pre_ represents the initial preoperative hematocrit (Hct) level, and Hct_post_ represents the postoperative Hct level; BV (L) = k_1_ × H^3^ + k_2_ × W + k_3_; for men, k_1_ = 0.3669, k_2_ = 0.03219, and k_3_ = 0.6041; for women, k_1_ = 0.3561, k_2_ = 0.03308, and k_3_ = 0.1833 (19–22). If an allogeneic transfusion or transfusion was performed, volume transfusions should be added when calculating total blood loss. Other postoperative outcomes included serum Hb level, change in Hb level, lower limb diameters on the first and third postoperative day (POD), ROM at discharge, length of postoperative hospital stay, and adverse events. A blood transfusion was used when the Hb concentration was less than 70 g/L or when a patient had any anemia-related complications, such as mental status changes or palpitation (Hb concentration between 7 and 10 g/dL) (17, 18). Thigh swelling was measured according to the change in lower limb diameters (2 points; thigh girth at 10 and 20 cm proximal to the patella superior border) (6). Adverse events were recorded until the last day of follow-up.

### Data analyses

The SPSS 22.0 software (IBM Corp.) was performed for data management and analysis. Continuous variables are described as means ± standard deviations and were analyzed using an independent Student’s *t*-test or a nonparametric test according to whether the data were normally distributed. Categorical variables are presented as numbers and percentages. Categorical variables were analyzed by Pearson chi-square test or Fisher’s exact test according to whether the expected frequencies were greater than five. The level of significance was set at *p* < 0.05.

## Results

**Figure 2:**
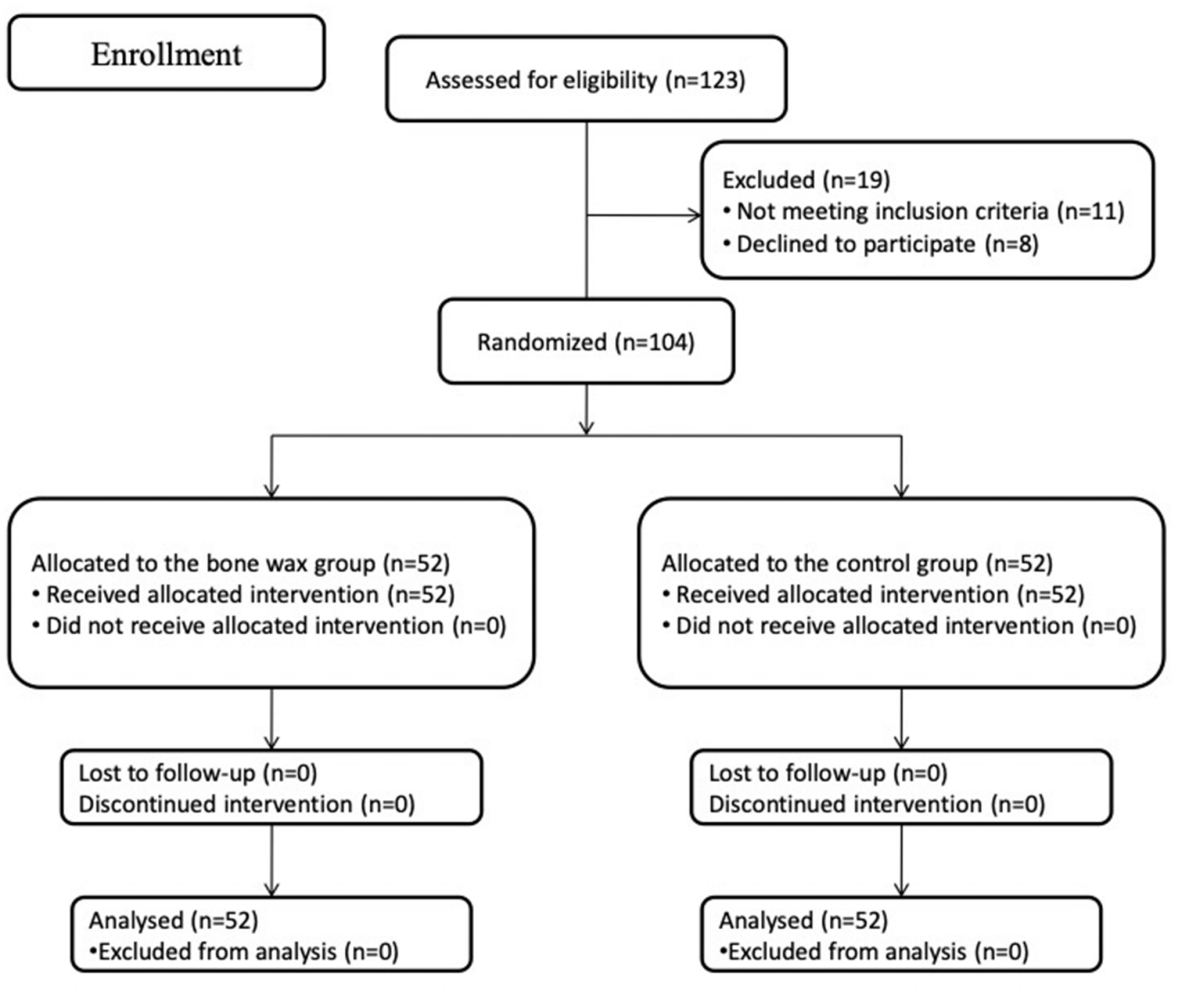
Schematic of the study design.

During the recruitment period from March 2021 to June 2021, 123 patients were scheduled to undergo primary unilateral THA because of end-stage joint diseases at our department. Among 123 patients, 11 patients did not meet the inclusion criteria, and eight patients declined to participate in the study. Therefore, the remaining 104 patients were eligible and formed the study cohort and were then assigned to either the bone wax group or the control group. The flow of patients is plotted in Figure 2. Preoperative demographics and characteristics were not significantly different between the groups (Table 1) (all *p* > 0.05). All participants included in the study were followed up for 6 months.

**Table 1:** Preoperative demographics and characteristics.

	Bone wax (*n* = 52)	Control (*n* = 52)	*P-*value
Demographic data			
Age* (yr)	55.1 ± 14.0	55.8 ± 15.6	0.802
Male sex^†^ [no. (%) of patients]	18 (34.6%)	23 (44.2%)	0.316
Height* (cm)	161.7 ± 9.3	160.4 ± 8.0	0.470
Weight* (kg)	62.6 ± 11.3	61.8 ± 9.5	0.694
BMI* (kg/m^2^)	23.9 ± 3.4	24.0 ± 3.5	0.842
ASA status^†^ [no. (%) of patients]			0.609
I	6 (11.5%)	9 (17.3%)	
II	33 (63.5%)	33 (63.5%)	
III	13 (25.0%)	10 (19.2%)	
Affected side (right/left)	23/29	27/25	0.432
Diagnosis^†^ (no. of patients)			0.769
Osteonecrosis of femoral head	19 (36.5%)	17 (32.7%)	
Primary osteoarthritis	16 (30.8%)	13 (25.0%)	
Developmental dysplasia of the hip	10 (19.2%)	12 (23.1%)	
Others	7 (13.5%)	10 (19.2%)	
Serum Hb level* (g/L)	137.4 ± 12.3	139.8 ± 12.3	0.359
Blood volume* (mL)	3917.4 ± 726.4	3890.7 ± 565.5	0.835
Preoperative parameters*			
Proximal thigh girth from the patella superior border			
10 cm	39.6 ± 4.0	40.2 ± 3.0	0.421
20 cm	46.9 ± 4.4	47.9 ± 3.7	0.239
Hip function			
Flexion (°)	91.4 ± 20.5	94.0 ± 15.7	0.470
Abduction (°)	26.0 ± 10.9	29.9 ± 9.8	0.055
Harris hip score (points)	41.1 ± 9.4	42.2 ± 10.3	0.578

*Data are given as the mean ± standard deviation. ^†^Data are given as the number (percentage) of patients.

Total blood loss, serum Hb level, and change in Hb level on PODs 1 and 3 in patients in the bone wax group were significantly better than those in the control group (Table 2) (*p* < 0.05). In terms of the risk of transfusion, two patients in the control group required transfusions, whereas none in the bone wax group required transfusion; however, the difference was not significant (Table 2) (*p* = 0.495). Moreover, patients in the bone wax group had a significantly shorter length of postoperative hospital stay than those in the control group (Table 2) (*p* = 0.002). No significant differences were observed in lower limb diameters on PODs 1 and 3, ROM at discharge, and Harris hip score 3 weeks and 6 months postoperatively. Regarding adverse events, one case of blood oozing from the wound occurred in the control group. No symptomatic thromboembolic events or other serious complications such as infection occurred in either of the groups.

**Table 2:** Postoperative outcomes.

	Bone wax (*n* = 52)	Control (*n* = 52)	*P*-value
Total blood loss* (mL)			
POD 1	249.4 ± 141.6	323.3 ± 154.3	*0.012*
POD 3	363.7 ± 153.8	450.0 ± 168.7	*0.008*
Serum Hb level* (g/L)			
POD 1	119.6 ± 11.3	114.5 ± 14.3	*0.045*
POD3	109.0 ± 12.4	102.8 ± 13.8	*0.018*
Change in Hb level* (g/L)			
POD 1	17.8 ± 8.8	25.3 ± 9.6	*<0.001*
POD 3	28.5 ± 11.7	37.0 ± 11.1	*<0.001*
Transfusion^†^ [no. (%) of patients]	0 (0.0%)	2 (3.8%)	0.495
Length of postoperative hospital stays* (h)	86.4 ± 15.0	97.8 ± 21.0	*0.002*
Postoperative parameters*			
10 cm above the patella superior border			
POD 1	40.3 ± 3.6	41.0 ± 3.0	0.333
POD 3	40.9 ± 3.6	41.6 ± 3.2	0.297
20 cm above the patella superior border			
POD 1	48.2 ± 4.2	48.9 ± 4.1	0.408
POD 3	49.1 ± 4.1	49.8 ± 4.0	0.330
Hip function at discharge			
Flexion (°)	102.1 ± 5.0	101.9 ± 4.4	0.836
Abduction (°)	38.8 ± 2.4	38.5 ± 2.9	0.581
Harris hip score (points)			
PO 3w	75.4 ± 5.3	73.8 ± 5.9	0.140
PO 6 m	87.2 ± 4.5	87.8 ± 4.2	0.449

PO, postoperative. *Data are given as the mean ± standard deviation. ^†^Data are given as the number (percentage) of patients. *p*-values indicating a significant difference among groups are in italics.

## Discussion

In this study, we found that the administration of bone wax effectively reduced postoperative total blood loss and changes in Hb levels on PODs 1 and 3. Moreover, patients in the bone wax group had better performance in terms of postoperative serum Hb level, change in Hb level on PODs 1 and 3, and length of postoperative hospital stay (all *p* < 0.05). The use of bone wax did not increase the incidence of bone wax-related adverse events.

The use of bone wax in orthopedic surgery has been well established in the literature, and many studies have demonstrated its efficacy in reducing blood loss and transfusion rate (7, 10). However, few studies have focused on the efficacy of bone wax in reducing postoperative blood loss after arthroplasty (6, 11). In a prospective randomized controlled study including 100 patients found that the application of bone wax was safe and effective for reducing total blood loss and maintaining higher hemoglobin levels (6). Another retrospective study about 674 consecutive surgeries declared the use of bone wax significantly reduced blood loss, decreased Hb levels, and the risk of transfusion (11). These results were substantiated by our study, however, these studies that have been conducted to determine the efficacy of bone wax in reducing blood loss following total joint replacement have focused on total knee arthroplasty (TKA) and not on THA. Because of the intrinsic difference between THA and TKA, a prospective randomized controlled study with a necessary sample size is appropriate and adequate to validate the effect of bone wax in reducing blood loss in THA. Thus, the effect of bone wax on THA remains unclear. Therefore, to resolve the aforementioned issue, we conducted a randomized controlled study to validate the effect of bone wax in reducing blood loss in THA.

During THA, after cancellous and cortical bones containing vascular tissues are incised, damage to the vasculature can cause osseous hemorrhage which is difficult to control by the natural hemostasis process and could result in severe blood loss, blood transfusions, and even mortality (23). Various surgical techniques have been proposed in different studies to effectively manage bleeding from the cortical and cancellous bone. Among these popular techniques, fibrin sealants require blood from the patient to obtain fibrin-rich cryoprecipitate, and its efficacy in reducing blood loss is heterogeneous in different studies (24). Collagen faces problems with storage stability, cohesiveness, and biocompatibility (10). Bone wax, with advantages such as ease of use during surgery, satisfactory cohesion to bone, malleability, and cost-effectiveness, is widely used for bone hemostasis (6).

Although bone wax has a long history of orthopedic application, as a foreign local agent, many orthopedists have raised concerns, such as failed bone healing, foreign body reaction, granuloma growth, thrombosis, and infection (10, 25–28). However, in the literature, the number of adverse events caused by bone wax is very small (28). Our results also demonstrated that bone wax did not increase the incidence of adverse events after THA.

This study had several limitations. First, this study was a single-center study, so the generalizability of the study may be influenced. However, many factors including individuals, centers, inclusion and exclusion criteria could also affect the generalizability. Second, diagnostic Doppler ultrasound and CT evaluation for symptomatic DVT and PE may miss asymptomatic venous thromboembolism. Finally, the necessary sample size estimation was calculated for our primary outcome, so this study may not be adequate to detect all relevant secondary outcomes.

## Conclusion

The administration of bone wax in THA effectively reduced total blood loss, decreased Hb level and length of postoperative stay, without increasing the incidence of adverse events. Therefore, bone wax shows potential and promise as a standard hemostasis agent and can be used for optimizing perioperative blood-conserving management protocols in THA.

## Data availability statement

The original contributions presented in the study are included in the article/supplementary material, further inquiries can be directed to the corresponding author.

## Ethics statement

The studies involving humans were approved by the Ethics Committee on Biomedical Research, West China Hospital of Sichuan University. The studies were conducted in accordance with the local legislation and institutional requirements. The participants provided their written informed consent to participate in this study.

## Author contributions

HL: study design and writing. CH: data collections and writing. Z-CD: data collections and data analysis. Z-HL: data collections and data analysis. E-ZZ: writing. Z-KZ: study design. All authors read and approved the final manuscript.

## Funding

This work was supported by 1.3.5 Project for Disciplines of Excellence, West China Hospital, Sichuan University (No. ZYJC18039), and Regional Innovation and Cooperation Program of Science and Technology Department of Sichuan Province (No. 2021YFQ0028).

## Conflict of interest

The authors declare that the research was conducted in the absence of any commercial or financial relationships that could be construed as a potential conflict of interest.

## Publisher’s note

All claims expressed in this article are solely those of the authors and do not necessarily represent those of their affiliated organizations, or those of the publisher, the editors and the reviewers. Any product that may be evaluated in this article, or claim that may be made by its manufacturer, is not guaranteed or endorsed by the publisher.

## Abbreviations

THA: total hip arthroplasty
ASA: American Society of Anesthesiologists
Hb: hemoglobin
DVT: deep vein thrombosis
PE: pulmonary embolism
CT: computed tomography
ROM: range of motion
POD: postoperative day
TKA: total knee arthroplasty
